# Bacteria of the genus *Rhodopseudomonas* (Bradyrhizobiaceae): obligate symbionts in mycelial cultures of the black truffles *Tuber melanosporum* and *Tuber brumale*

**DOI:** 10.1186/s40064-016-2756-6

**Published:** 2016-07-15

**Authors:** Christine Le Roux, Estelle Tournier, Adrien Lies, Hervé Sanguin, Gérard Chevalier, Robin Duponnois, Daniel Mousain, Yves Prin

**Affiliations:** CIRAD, UMR LSTM, 34398 Montpellier Cedex 5, France; INRA Centre de Recherche de Clermont-Theix, 63039 Clermont-Ferrand Cedex, France; IRD, UMR LSTM, 34398 Montpellier Cedex 5, France; Société d’Horticulture et d’Histoire Naturelle de l’Hérault, Parc à Ballon 1, bâtiment B, 125 rue du Moulin de Sémalen, 34000 Montpellier, France

**Keywords:** Truffle, Ascomycete, Procaryote, In vitro production, Cultivability

## Abstract

**Background:**

This work aimed at characterizing 12 isolates of the genus *Tuber* including *Tuber melanosporum* (11 isolates) and *Tuber brumale* (one isolate). This was done using internal transcribed spacer (ITS) sequences, confirming their origin.

**Results:**

Analysis of their mating type revealed that both *MAT1*-*1* and *MAT1*-*2* exist within these isolates (with 3 and 8 of each, respectively). We observed that each of these cultures was consistently associated with one bacterium that was intimately linked to fungal growth. These bacterial associates failed to grow in the absence of fungus. We extracted DNA from bacterial colonies in the margin of mycelium and sequenced a nearly complete 16S rDNA gene and a partial ITS fragment. We found they all belonged to the genus *Rhodopseudomonas*, fitting within different phylogenetic clusters. No relationships were evidenced between bacterial and fungal strains or mating types. *Rhodopseudomonas* being a sister genus to *Bradyrhizobium*, we tested the nodulation ability of these bacteria on a promiscuously nodulating legume (*Acacia mangium*), without success. We failed to identify any *nif*H genes among these isolates, using two different sets of primers.

**Conclusions:**

While the mechanisms of interaction between *Tuber* and *Rhodopseudomonas* remain to be elucidated, their interdependency for in vitro growth seems a novel feature of this fungus.

**Electronic supplementary material:**

The online version of this article (doi:10.1186/s40064-016-2756-6) contains supplementary material, which is available to authorized users.

## Background

Truffles, hypogeous ascomycetes belonging to the genus *Tuber*, include ectomycorrhizal species of major socioeconomic interest. Some species, such as *Tuber melanosporum*, *Tuber magnatum* and *Tuber aestivum*, are edible and have great market value. Production of truffles depends on tree saplings (of species belonging to genera such as *Quercus*, *Corylus* and *Tilia*) appropriately inoculated with fungal inoculants, produced within traditional or industrial nurseries. Various forms of inocula may be used in these nurseries, ranging from soil under productive trees, through crushed fresh, deep frozen or dried fruit bodies (whole or as debris), to mycelial cultures. Several authors have shown that it is possible to synthesize mycorrhizas with *Tuber* mycelial cultures (Chevalier and Frochot 1997; Sisti et al. 1998). However, *Tuber* species are generally difficult to isolate and cultivate in laboratory conditions, species like *Tuber borchii* (Barbieri et al. 2005), *Tuber rufum*, *Tuber uncinatum* and *Tuber macrosporum* (Iotti et al. 2002) being among the easiest.

At least for two species (*T. magnatum* and *T. melanosporum*) the long-standing question of whether *Tuber* species are homo- or heterothallic was recently solved with the identification of two mating type loci carrying either *MAT1*-*1*-*1* or *MAT1*-*2*-*1* genes (Rubini et al. 2011). *MAT1*-*1*-*1* and *MAT1*-*2*-*1* encode a protein with an alpha domain, and a high-mobility DNA binding protein (HMG), respectively. In these species, sexual reproduction, which is necessary for fructification, only occurs between two different mating types. In fructification, each spore is of only one mating type, and the proportion of spores of each mating type is about 50 %. The gleba is from only one mating type, identical to that of all the ectomycorrhiza surrounding the fructification (Rubini et al. 2011). Mating type identification is thus a major challenge on the way to mastering *Tuber* fructification in the soil. Strain Mel28 of *T. melanosporum*, whose genome has been fully sequenced (Martin et al. 2010), is of the *MAT1*-*2*-*1* type. Having mycelial cultures representative of different mating types would be of great interest both for lab experiments and plant tests.

Bacteria are known to be ubiquitously associated with ectomycorrhization (for the concept of mycorrhiza helper bacteria, see the review by Frey-Klett et al. 2007) and neither truffle ectomycorrhiza nor their fruit bodies (Table 1) are exceptions. Recently, Mello et al. (2013) showed that “brûlés”—burnt areas around productive trees—of *T. melanosporum* markedly affected soil bacterial communities. These bacteria may have various effects on *Tuber* mycelium growth, including inhibition and promotion. For example, one strain of *Staphylococcus aureus* has been shown to produce volatile organic compounds potentially involved in *T. borchii* mycelial growth inhibition (Barbieri et al. 2005). Unexpected procaryotic functional activities like nitrogen fixation have been evidenced in ascocarps of *T. magnatum* by Barbieri et al. (2010). Bacteria have been shown to participate in truffle aroma elaboration through the production of thiophene volatiles (Splivallo et al. 2015). In a recent paper, Benucci and Bonito (2016) observed by 454 pyrosequencing the dominance of the genus *Bradyrhizobium* within *Tuber* ascocarps of various geographic origins, but not in other truffle genera like *Kalapuya*, *Terfezia* or *Leucangium*.

**Table 1:** Diversity of bacterial genera characterized either directly (“ascocarps”) or after isolation from ascocarps (“isolates”) or mycelial cultures of different *Tuber* species in recent publications

*Tuber* ascocarp	Dominant bacterial genera	Bacterial DNA origin	References
*T. aestivum*	*Pseudomonas, Raoultella*	Isolates	Rivera et al. (2010)
*T. borchii*	*Cytophaga/Flexibacter/Bacteroides* group	Mycelial cultures	Barbieri et al. (2000)
*T. borchii*	*Bradyrhizobium,* rhizobia *s.l., Pseudomonas*	Isolates and ascocarps	Barbieri et al. (2005)
*T. borchii*	*Pseudomonas*	Isolates	Bedini et al. (1999)
*T. borchii*	*Pseudomonas fluorescens,* spore-forming Bacillaceae	Isolates	Citterio et al. (2001)
*T. borchii*	*Pseudomonas* spp	Isolates	Sbrana et al. (2000)
*T. gibbosum*	*Bradyrhizobium, Rhizobium,* etc.	Ascocarps	Benucci and Bonito (2016)
*T. indicum*	*Bradyrhizobium, Methylibium,* etc.	Ascocarps	Benucci and Bonito (2016)
*T. lyonii*	*Bradyrhizobium, Polaromonas,* etc.	Ascocarps	Benucci and Bonito (2016)
*T. magnatum*	*Sinorhizobium,* rhizobia *s.l., Bradyrhizobium, Pseudomonas*	Isolates and ascocarps	Barbieri et al. (2007)
*T. magnatum*	*Bradyrhizobium*	Ascocarps	Barbieri et al. (2010)
*T. melanosporum*	*Bradyrhizobium, Bacteroidetes* (peridium)	Ascocarps	Antony-Babu et al. (2013)
*T. melanosporum*	*Bradyrhizobium, Polaromonas,* etc.	Ascocarps	Benucci and Bonito (2016)
*T. melanosporum*	*Pseudomonas, Enterobacter*	Isolates	Rivera et al. (2010)
*T. oregonense*	*Bradyrhizobium, Methylibium,* etc.	Ascocarps	Benucci and Bonito (2016)

However, information is still lacking on the characteristics of mycelial cultures of *T. melanosporum*, including their associated bacteria. The aim of this study is to identify the bacterial strains associated with *T. melanosporum* and *T. brumale* in culture, as it could help to control mycelial isolation, to mass produce *Tuber* inoculum and to generate truffle productive saplings.

## Methods

### Isolation and culture of *Tuber* mycelium

Mycelia were originally isolated from ethanol-sterilized fruit bodies of *T. melanosporum* and *T. brumale* (Table 2), by axenically placing a piece of gleba on solid Maltea Moser medium, according to Chevalier (1972). All isolates were routinely subcultured on 2 % Cristomalt^®^ (Difal, Seysses, France) agar medium (modified from Chevalier (1972), Maltea Moser being replaced by Cristomalt), at 25 °C in the dark. When necessary the antibiotics chloramphenicol, tetracycline, gentamicin and streptomycin were individually added to the medium at concentrations routinely used in the lab for ectomycorrhizal mycelium cultivation, i.e., 50, 10, 10, and 80 mg l^−1^, respectively (Bâ et al. 2011).

**Table 2:** List of the *Tuber* mycelial cultures used in this study, with their associated host and geographical origin

Strain	*Tuber* ascocarp	Original host^a^	Site	Author^b^	Date
MelBal1	*T. melanosporum*	*Corylus avellana* (WT)	Troussey, Meuse, France	C.D.	February, 1988
MelBal3	*T. melanosporum*	*C. avellana* (WT)	Troussey, Meuse, France	C.D.	February, 1988
BTR3	*T. melanosporum*	*Quercus pubescens* (O)	INRA, Clermont-Fd, France	A.O.	December, 1999
MelCR2-00	*T. melanosporum*	*Q. pubescens* (O)	INRA, Clermont-Fd, France	A.O	January, 2000
MelC89	*T. melanosporum*	nd	*leg.* INRA Bordeaux	nd	nd
Mel2VDA3	*T. melanosporum*	*Quercus sp.* (WT)	Andryes, Yonne, France	A.O.	February, 1998
Mel3VDA4	*T. melanosporum*	*Q. sp.* (WT)	Andryes, Yonne, France	A.O.	February, 1998
Mel14	*T. melanosporum*	*Quercus ilex* (WT)	Apt,Vaucluse, France	C.D.	February, 1988
Mel18	*T. melanosporum*	nd	INRA Coulaures, Dordogne, France	A.O	January,1994
Mel28	*T. melanosporum*	*Q. ilex* (WT)	Maillane, Bouches-du-Rhône, Fr.	C.D.	February, 1988
MelBaud1	*T. melanosporum*	*Q. pubescens* (O)	Bauduen, Var, France	C.D.	March, 1998
TBRS	*T. brumale*	*Q. pubescens* (O)	Charente, France	J.T.	1991

*nd* not determined

^a^ *WT*: Wild truffle, *O* Orchard

^b^ *C. D*. C. Dupré, *A.O.* A. Oudin, *J.T.* J. Tourvieille

Bacterial strain cultivation assays were attempted on yeast mannitol agar (YMA) medium (Vincent 1970), classically used for cultivating *Bradyrhizobium* and *Rhodopseudomonas* in the lab. Bacterial strains were named by placing B before the number of the fungal strain with which they were associated (e.g., BMel18 for the bacteria associated with the fungal strain Mel18).

### Microscopic observations

Changes in mycelial and bacterial growth were followed in Petri dishes examined with a Nikon AZ100 microscope.

### Molecular characterization

Total fungal DNA was extracted using REDExtract-N-Amp polymerase chain reaction (PCR) kits (Sigma-Aldrich, St. Louis, MO) according to the manufacturer’s instructions. Mycelium was confirmed as *Tuber* by analysis of internal transcribed spacer (ITS) sequences using the highly conserved fungal rRNA gene primers ITS1F (Gardes and Bruns 1993) and ITS4 (White et al. 1990) for PCR. Each PCR reaction (25 µl) contained 2 µl of template DNA, 1× Reaction Buffer (1.5 mM MgCl_2_), 200 µM of each dNTP, 0.5 µM of each primer, 2× bovine serum albumin and 1 U of GoTaq^®^ DNA polymerase (Promega Corporation, Madison, Wi). The PCR thermal protocol consisted of an initial 5 min denaturation step at 95 °C, 35 amplification cycles of 95 °C for 30 s, 52 °C for 1 min, 72 °C for 1 min, and a final extension step of 72 °C for 10 min. After agarose gel electrophoresis, gel bands of the expected size were excised and PCR products were purified using Illustra GFX PCR DNA and Gel Band Purification Kit (GE Healthcare, UK). DNA was sequenced (Genoscreen, France) with the same primer ITS1F as used for PCR. Complementarily, *Tuber* mating types were determined by PCR with each of the pairs of primers dedicated to *MAT1*-*1* and *MAT1*-*2* according to Rubini et al. (2011).

Bacteria were characterized according to their nearly complete 16S rDNA sequences and their partial 16S-23S rRNA ITS. A loopful of bacterial cells, taken from the margin of the mycelial colony, was suspended in 20 µl of sterile water and cell debris removed by centrifugation at 13,000 rpm for 1 min at room temperature; 2 µl of the supernatant was used as a template for PCR.

Amplification of the nearly complete 16S rDNA was performed for each bacterial strain using forward (FGPS6 5′-GGAGAGTTAGATCTTGGCTCAG-3′) and reverse (FGPS1509 5′-AAGGAGGGGATCCAGCCGCA-3′) primers (Normand et al. 1992). Each PCR amplification was carried out in a 50-µl reaction tube containing 4 µl of bacterial DNA template, 1× Reaction Buffer (1.5 mM MgCl_2_), 200 µM of each dNTP, 0.8 µM of each primer, and 1.25 U of GoTaq^®^ DNA polymerase (Promega Corporation, Madison, Wi), with the following temperature cycles: an initial cycle of denaturation at 96 °C for 3 min; 35 cycles of denaturation at 95 °C for 30 s, annealing at 55 °C for 30 s, and extension at 72 °C for 1 min; and a final extension at 72 °C for 3 min. The PCR products were directly sequenced using the same primers as for amplification, FGPS6 and FGPS1509, and primer 16S-1080r (5′-GGGACTTAACCCAACATCT-3′; Sy et al. 2001). Sequencing was performed by Genoscreen (Lille, France).

The partial ITS of the 16S and 23S rRNA genes was amplified using primers BR5 (5′-CTTGTAGCTCAGTTGGTTAG-3′; Willems et al. 2001) and FGPL132′ (5′-CCGGGTTTCCCCATTCGG-3′; Ponsonnet and Nesme 1994). Each PCR amplification was carried out in a 25-µl reaction tube containing 2 µl of bacterial DNA template, 1× Reaction Buffer (1.5 mM MgCl_2_), 200 µM of each dNTP, 0.8 µM of each primer, and 0.62 U of GoTaq^®^ DNA polymerase (Promega Corporation, Madison, Wi). The PCR thermal protocol was as described in Le Roux et al. (2014). The PCR products were directly sequenced using the same primer BR5. Sequencing was performed by Genoscreen (Lille, France).

For *nif*H genes, two pairs of primers were tested, nifHF/nifHI (Laguerre et al. 2001) and polF/polR (Poly et al. 2001). They were tested on four randomly chosen bacteria: BMel18, BMel28, BmelC89 and BBTR3. The PCR mix and thermal conditions were as described earlier except for the annealing conditions: 57 °C for 1 min with nifHF/nifHI and 55 °C for 30 s with polF/polR. A positive control was performed using *Bradyrhizobiumdiazoefficiens* Type strain USDA 110 originally isolated from soybean nodule in Florida, in 1957 (Delamuta et al. 2013).

### Nucleotide sequence analyses

Fungal ITS, bacterial 16S rRNA and ITS 16S-23S rRNA sequences were corrected using the sequence viewer program 4 Peaks (http://4peaks.en.softonic.com/mac, accessed 11 March 2016). The fungal ITS, bacterial 16S rRNA and ITS sequences were deposited in GenBank and their accession numbers are presented in Tables 3 and 4. For bacterial characterization, multiple alignment and phylogenetic tree construction were performed using the multiplatform program SeaView version 4 (Gouy et al. 2010). This interface drives the Clustal Omega program for multiple sequence alignments and includes the BioNJ distance-based tree reconstruction method and the maximum likelihood (ML) based phylogeny program PhyML.

**Table 3:** Molecular characterization (partial ITS sequencing) and mating types of *Tuber* spp. mycelial cultures

Mycelial culture	Sequence length (bp)	Accession no	Closest BLASTn (accession no)	Identity	Mating type
BTR3	603	KM659869	*T. melanosporum* (AF132501)	100	MAT 1-2-1
Mel14	nd	nd	nd	nd	MAT 1-1-1
Mel18	591	KM659866	*T. melanosporum* (GU979083)	100	MAT 1-2-1
Mel28	550	KM659874	*T. melanosporum* (GU979083)	100	MAT 1-2-1
Mel2VDA3	604	KM659868	*T. melanosporum* (GU979083)	100	MAT 1-2-1
Mel3VDA4	598	KM659867	*T. melanosporum* (GU979083)	100	MAT 1-1-1
MelBal1	604	KM659870	*T. melanosporum* (AF300826)	99	MAT 1-2-1
MelBal3	571	KM659873	*T. melanosporum* (GU810153)	99	MAT 1-2-1
MelC89	609	KM659871	*T. melanosporum* (GU979083)	99	MAT 1-1-1
MelCR2-00	610	KM659872	*T. melanosporum* (GU810153)	99	MAT 1-2-1
MelBaud1	nd	nd	nd	nd	nd
TBRS	880	KM659875	*T. brumale* (JF926118)	99	MAT 1-2-1

*nd* not determined

**Table 4:** Molecular characterization (near full 16S rDNA and partial ITS) of bacteria associated with *Tuber* spp. mycelial cultures

Bacterial isolate	16S sequence length (bp)	Accession no	Closest BLASTn (accession No)	Identity (%)	ITS sequence length (bp)	Accession no	Closest BLASTn (accession No)	Identity (%)
BBTR3	1358	KM597510	*Rhodopseudomonas* sp. (AJ968691)	100	784	KM597522	*Bradyrhizobium* sp. (EU288750)	96
BMel14	601	KM597518	*Rhodopseudomonas* sp. (KF663061)	99	nd	nd	nd	nd
BMel18	1358	KM597517	*Rhodopseudomonas* sp. (AJ968691)	100	782	KM597527	*Bradyrhizobium* sp. (EU288750)	96
BMel28	1358	KM597515	*Rhodopseudomonas* sp. (AJ968691)	100	784	KM597525	*Bradyrhizobium* sp. (EU288750)	96
BMel2VDA3	1358	KM597512	*Rhodopseudomonas* sp. (AJ968691)	100	771	KM597528	*Bradyrhizobium* sp. (EU288750)	93
BMel3VDA4	1353	KM597507	*Rhodopseudomonas* sp. (AJ968691)	99	767	KM597519	*Bradyrhizobium* sp. (EU288750)	90
BMelBal1	1356	KM597513	*Rhodopseudomonas* sp. (KF663061)	99	761	KM597529	*Bradyrhizobium* sp. (EU288750)	83
BMelBal3	1329	KM597508	*Rhodopseudomonas* sp. (KF663061)	99	759	KM597520	*Bradyrhizobium* sp. (EU288750)	83
BMelC89	1356	KM597511	*Rhodopseudomonas* sp. (KF663061)	99	765	KM597523	*Bradyrhizobium* sp. (EU288750)	83
BMelCR2-00	1406	KM597514	*Rhodopseudomonas* sp. (KF663061)	99	759	KM597524	*Bradyrhizobium* sp. (EU288750)	83
BMelBaud1	1333	KM597509	*Rhodopseudomonas* sp. (AJ968691)	100	771	KM597521	*Bradyrhizobium* sp. (EU288750)	93
BTBRS	1358	KM597516	*Rhodopseudomonas* sp. (AJ968691)	100	768	KM597526	*Bradyrhizobium* sp. (EU288750)	93

*nd* not determined

### Plant Nodulation test

Monoxenic nodulation assays were performed on *Acacia mangium* (a promiscuously nodulating legume) according to Perrineau et al. (2011) with four randomly chosen bacteria: BMelC89, BMelCR2-00, BMel3VDA4 and BMel18.

## Results

**Fig. 1:**
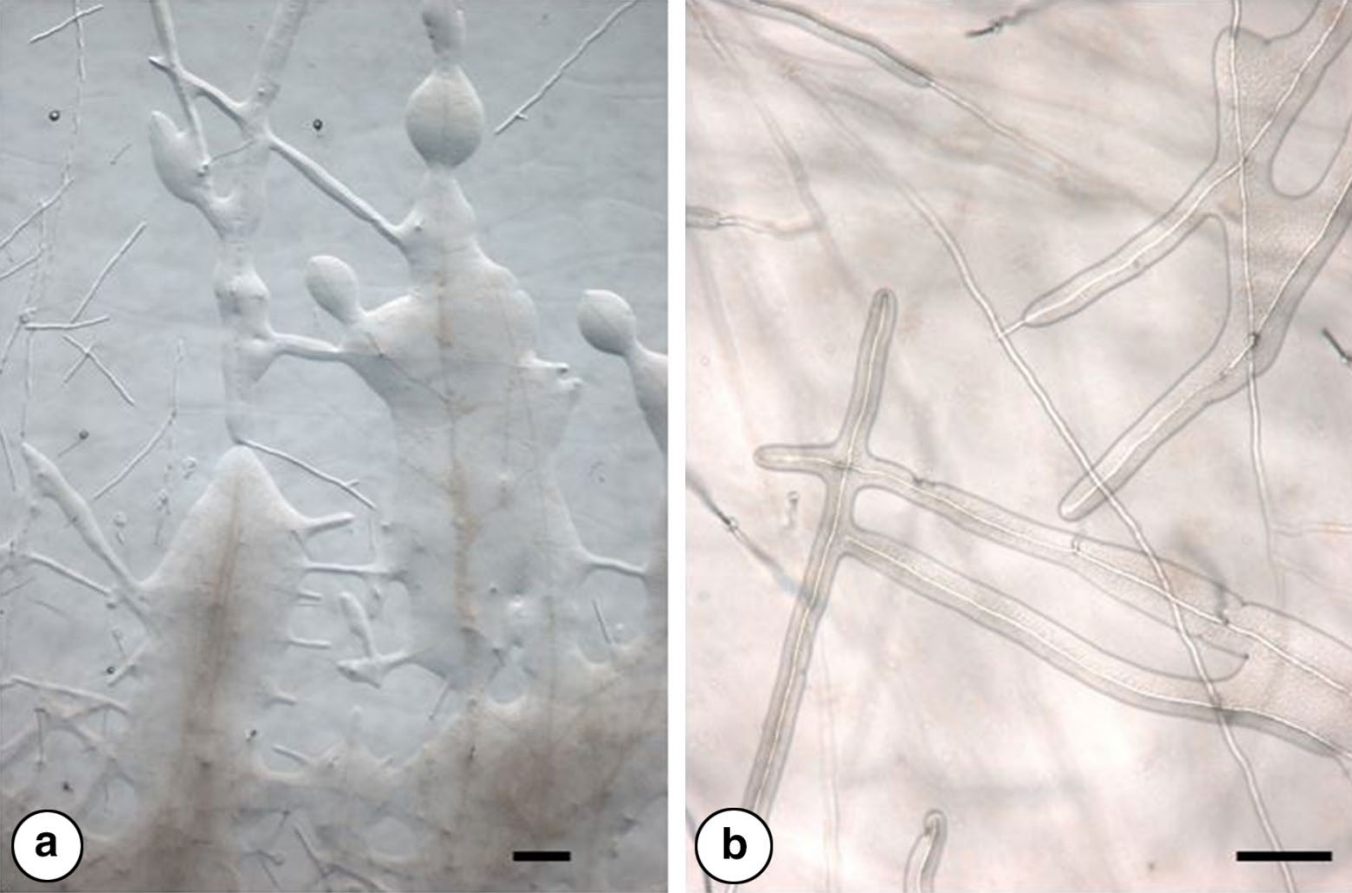
Microscopic observation of the bacterial colonization of growing hyphae of *Tuber melanosporum* isolate Mel18 by *Rhodopseudomonas* sp., on solid medium. **a** General view of the peripheral mycelia and the bacterial colonies. *Bar* is 100 µm. **b** progressive ensheathment of growing hyphae by *Rhodopseudomonas* sp. *Bar* is 50 µm

All isolates of *T. melanosporum* and *T. brumale* examined in the present study exhibited slow growth. They required 2–3 weeks to initiate a new, visible mycelium crown around the plug. The growth rate appeared to be slightly accelerated when regularly subcultured. Slow growth is a general feature of the genus *Tuber*, depending on the media, which are generally based on malt or potato dextrose agar. On these solid media, growth generally takes 4–8 weeks to stabilize at its maximum level. In our conditions, the average radial mycelial growth was estimated as 1 cm in 3 weeks. All the *Tuber* cultures included bacterial associates. The addition of chloramphenicol or tetracycline to the Cristomalt agar medium had little effect on bacterial (and mycelial) growth. Others antibiotics such as gentamicin or streptomycin totally blocked both mycelial and bacterial development. These bacterial isolates generally grew very poorly on YMA media, once isolated from *Tuber* mycelia, and did not survive repeated subculturing, limiting the possibilities of enzymatic or antibiotic resistance characterization. Under the microscope, mycelium generally appears as the first medium colonizer, outgrowing from the plug, the bacterial associate proliferating around growing hyphae and ensheathing them with a slight delay (Fig. 1a, b).

As presented in Table 3, fungal ITS sequencing confirmed the original taxonomic identity of each of the cultivated *Tuber* isolates. The mating types of the different mycelial cultures (Table 3) are presented in Additional file 1, three and eight of them being of the *MAT1*-*1* and *MAT1*-*2* type, respectively. The mating type being generally determined by PCR response to each pair of primers, not followed by sequencing, we tested these primers on some of the bacterial DNA extracts (BMelBal3, BMelC89, BMel2VDA3 and BMel3VDA4): none of them allowed us to obtain an amplicon. The PCR positive controls performed on fungal DNA representative of the two mating types (*T. melanosporum* strains MelC89 and Mel2VDA3 for *MAT1*-*1* and *MAT1*-*2*, respectively) were all positive, with a band of the expected size (421 and 550 bp for *MAT1*-*1* and *MAT1*-*2*, respectively; not illustrated).

**Fig. 2:**
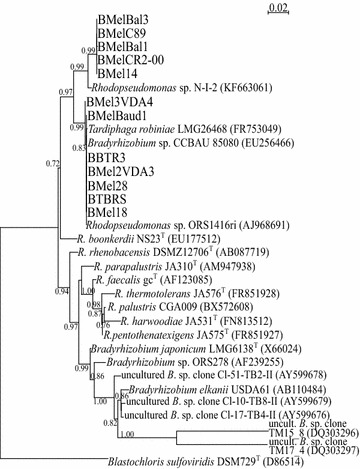
PhyML phylogenetic tree based on nearly complete 16S rRNA (1330 bp) sequences of 12 *Tuber* spp. associated bacterial strains aligned with *Rhodopseudomonas* spp. type strains (^T^) and related strains, including uncultured *Bradyrhizobium* sp. clones from *Tuber borchii* and *Tuber magnatum* ascocarps. Only branch support probabilities (estimated with the approximate likelihood-ratio test) higher than 0.70 are given at the branching points. Gaps were not considered. Scale indicates 2 % sequence divergence. *Blastochloris sulfoviridis* was chosen as an outgroup

The homologies of the nearly full-length 16S rRNA sequences of 11 bacterial isolates and one partial 16S rRNA sequence for BMel14 are presented in Table 4. All these sequences were close to *Rhodopseudomonas* spp. (Table 4) with an identity percentage of 99–100 %. The phylogenetic tree (Fig. 2) allows us to identify a first cluster of five strains closely related to *Rhodopseudomonas* sp. strain N-I-2, an endophytic bacteria isolated from *Prunus avium* (Quambush et al. 2014). The second cluster includes the seven other bacterial isolates and the strains *Rhodopseudomonas* sp. strain ORS1416ri (with 99–100 % identity), *Bradyrhizobium* sp. CCBAU 85080 and *Tardiphaga robiniae* LMG26468. The sequences of five bacterial strains from white truffles (*T. borchii* and *T. magnatum*; Barbieri et al. 2005, 2007) were also included in this phylogeny. They were selected as representative of the different 16S clusters obtained by these authors. They appear to be close to *Bradyrhizobium* strains, *B. elkanii* for *T. borchii*, and in a separate cluster for *T. magnatum*. Within the Bradyrhizobiaceae, all these associates of white truffle ascomata were quite distinct from our black truffle mycelial associates (Fig. 2).

**Fig. 3:**
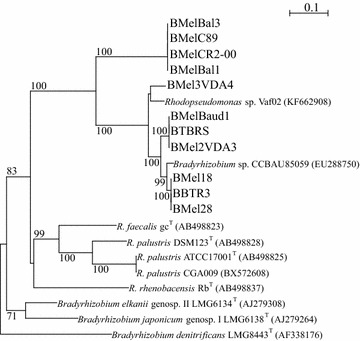
BioNJ phylogenetic tree based on 16S-23S rRNA ITS sequences of 11 *Tuber* spp. associated bacterial strains aligned with *Rhodopseudomonas* spp. type strains (^T^) and related strains. Only bootstrap probability values higher than 70 % (100 replicates) are given at the branching points. Gaps were not considered. Scale indicated 10 % sequence divergence. *Bradyrhizobium denitrificans* was chosen as an outgroup

The mean length of the partial 16S-23S rRNA ITS sequence was 770 bp. BLASTn analysis (Table 4) and phylogenetic tree reconstruction (Fig. 3) confirmed that all mycelial bacteria clustered within the *Rhodopseudomonas* clade, which also included the strain *Bradyrhizobium* sp. CCBAU 85059.

The trials that we carried out to test symbiotic characteristics such as nitrogen fixation and nodulation on our *Tuber* associated strains, remained unsuccessful: both pairs of *nifH* primers that we tested repeatedly failed to give a PCR product related to a *nifH* gene. The positive control with *B. diazoefficiens* type strain gave a band of the expected size with both pairs of primers. None of the bacterial strains nodulated the promiscuous legume *A. mangium* 3 months after inoculation. In these routinely used culture conditions, nodulation is known to usually occur within 2–3 weeks after inoculation (Perrineau et al. 2011).

## Discussion

As reported by Iotti et al. (2002), first isolation from inner ascocarp tissue is generally not too difficult as compared with subsequent subculturing, as many cultures do not survive that step, a phenomenon that had already been mentioned by Chevalier (1972). Both these papers also reported lag phases of different duration according to the species, with no relationships, regarding these lag phases, between the first outgrowth and the subsequent subculturing. They also reported that optimal culture media for isolating and subculturing could be different: casein hydrolysate and Maltea Moser, respectively (Chevalier 1972); or modified Woody Plant Medium and Malt or Potato Dextrose Agars, respectively (Iotti et al. 2002). Among important factors that may influence growth are pH, temperature and dietary elements (Michaels 1982, https://ir.library.oregonstate.edu/xmlui/handle/1957/9224, accessed 11 March 2016), most optimal temperatures being around 20 °C, and pH over 7. In such conditions, maximum growth was reached after 7 weeks for *T. melanosporum*, with marked intraspecific variations. This species was one of the slowest among the six tested by this last author. Cultures in liquid media have sometime been used: the only *Tuber* genome sequenced to date was obtained from liquid-grown mycelium of the strain Mel28 (Martin et al. 2010).

Based on ribosomal sequence analyses, bacterial associates were all found to belong to *Rhodopseudomonas*, a genus of alpha proteobacteria closely related to *Bradyrhizobium* (Giraud and Fleischman 2004). Among *Rhodopseudomonas* species, *R. palustris* is a photoautotrophic bacterium, taxonomically close to some photoheterotrophic *Bradyrhizobium* species, that efficiently nodulates stems and roots of the legume *Aeschynomene*. At this time, no strains of *R. palustris* are described as nodulating legumes, but they quite commonly harbor *nifH* genes (Cantera et al. 2004). The low discriminatory power of 16S rRNA has long been recognized within the Bradyrhizobiaceae (Willems et al. 2001), necessitating a complementary characterization using other targets, such as 16S-23S ITS. Both these targets allowed us to confirm the close relationships between *Rhodopseudomonas* and *Bradyrhizobium*. Moreover, when we re-blasted the ITS sequences of *Bradyrhizobium* sp. CCBAU 85059, isolated, as strain CCBAU 85080, from *Astragalus tatsienensis* nodules in Tibet (Hou et al. 2009), the closest identified strains belonged to the genus *Rhodopseudomonas*. This is probably a result of the fact that these authors only considered the genus *Bradyrhizobium* in their phylogenetic analyses. Concerning strain R_45974 of *T. robiniae*, isolated from root nodules of *Robinia pseudoacacia*, it was previously described as *Rhodopseudomonas* sp. (De Meyer et al. 2011) on the basis of a 16S rRNA gene phylogeny. More recently, it has been re-assigned to this new genus in Bradyrhizobiaceae after complementary characterization comprising physiological and biochemical tests, and sequencing of housekeeping genes (De Meyer et al. 2012).

More generally regarding molecular characterizations, no particular clustering of the bacterial sequences was detected in regard to our black truffle ascocarp species or geographical origin, mycelial strain or mating type: there is not, at this stage, any evidence of specificity between a given *Tuber* mycelium and its associated *Rhodopseudomonas*. However, the fact that, despite a relatively heterogeneous geographic origin of the ascocarps, all the mycelia-associated strains fall within the same genus *Rhodopseudomonas* is consistent with a non-random association. Similarly, the existence of several different clades within *Rhodopseudomonas* sequences (Figs. 2, 3) seems to exclude the hypothesis of an accidental contamination of all the mycelial cultures during successive subcultivation.

On the fungal side, according to Chevalier (1972) elimination of the mycelial bacteria frequently leads to the loss of the corresponding *T. melanosporum* culture. This author reported that one of these bacteria was attributable to the genus *Arthrobacter*, with the identification tools available at that time. More recently, Barbieri et al. (2000, 2002) showed that *T. borchii* mycelial cultures were associated with unculturable bacteria of the *Cytophaga*–*Flexibacter*–*Bacteroides* phylum. Remarkably, some of these bacteria were detected as viable within the hyphae. While the presence of other gram negative bacteria in mycelial cultures has already been reported, few or none of these associates were precisely identified.

Such studies to characterize the dependency of these *Tuber*–bacteria associations are made extremely difficult owing to (1) the slow growth of *Tuber* mycelium and (2) the non-cultivability of associated *Rhodopseudomonas* and thus difficulties in characterizing and eliminating bacteria from mycelial cultures.

Our *Tuber* isolates originating from ascocarp inner tissue, it seems likely that in vitro mycelia-accompanying bacteria could also be of ascocarpic origin. Although, after several decades, the original ascocarpic material is no longer available to check the identity of mycelium-associated strains to original ascocarpic bacteria, we know from the literature that truffle ascocarps have been shown to host a number of different microbes including yeasts (Buzzini et al. 2005), fungi (Pacioni et al. 2007) and bacteria (Table 1). Some bacterial genera have repeatedly been reported as colonizing ascocarpic tissue of various *Tuber* species (Table 1). They belonged to different lineages of proteobacteria, most of them being within alpha or gamma proteobacteria. Among the dominant genera, *Bradyrhizobium* and *Pseudomonas* were almost universally reported whatever the *Tuber* species under consideration and be it after isolation or directly from total ascocarp DNA. It has to be noted that bacterial communities varied according to the degree of maturation of the ascocarp as shown with *T. melanosporum* (Antony-Babu et al. 2013) and *T. magnatum* (Barbieri et al. 2007). *Bradyrhizobium* is over-represented within bacterial communities directly characterized from the ascocarp, as is the case for *Pseudomonas* among bacterial isolates. In *T. magnatum* ascocarp, Barbieri et al. (2010) reported significant amounts of nitrogen fixation (nitrogenase activity estimated by acetylene reduction), together with the presence of *nifH* genes in ascocarps at different degrees of maturation. The phylogenetic positions of bradyrhizobia associated with ascocarps of *T. borchii* (Barbieri et al. 2005) and *T. magnatum* (Barbieri et al. 2007) showed that none of them clustered with our *T. melanosporum* or *T. brumale* mycelial bacteria. As Antony-Babu et al. (2013) remind us, *Bradyrhizobium* is “consistently found at all stages of the maturation process and in different truffle species”. These authors suggested that the selection of Bradyrhizobiaceae results from deterministic events allowing these bacterial taxa to tolerate and colonize the particular environment of the ascocarpic tissues (with sulfur-containing molecules and aromatic volatile compounds).

During the isolation steps, two main pitfalls have to be avoided: the duration of hyphal outgrowth from the *Tuber* explant in competition with other ascocarp inhabiting microbes, and failure to grow after the first subculture, a particular fate reported by Giomaro et al. (2005). These authors argue that this could be due to the non-acclimation of the ascocarpic mycelium to the saprophytic stage of in vitro growth. A positive role of the *Tuber* associated bacterial strains as helpers in this progressive adaptation to the new lifestyle cannot be excluded. However, in the absence of pure, bacteria-free cultures of *Tuber* mycelium, all the confrontation trials (co-cultivation of *Rhodopseudomonas*-associated mycelium and freshly isolated *Rhodopseudomonas*, on various solid media) we attempted (data not shown) were inconclusive in terms of fungal growth response (radial mycelial growth).

In this study, we observed that whatever the origin of the ascocarp, none of the 12 mycelial cultures was devoid of *Rhodopseudomonas* associates. However, these associates are genetically diversified in several clusters, for both 16S and ITS rDNA, without evidencing any relationship between strain clustering and criteria such as geographical origin of ascocarps, age of mycelial culture since its isolation (27–15 years), isolation operator, mycelium mating type or *Tuber* species. In a separate experiment, conducted in 2013 to isolate bacterial associates from fresh *T. borchii* and *T. melanosporum* ascocarps, we never obtained *Rhodopseudomonas* strains among 123 and 126 bacterial isolates from each ascocarp, respectively (data not shown), which seems in accordance with non-cultivability of these *Tuber* associates. Remarkably, Bradyrhizobiaceae are often well represented among ascocarpic DNA, whatever the *Tuber* species, but absent from isolates in the different studies listed in Table 1, the only exception being one isolate from *T. magnatum* that grouped with bacteria of the genus *Bosea*, another member of the Bradyrhizobiaceae (Barbieri et al. 2007). Attempts to separate mycelium from bacteria (on several selected antibiotics) remained unsuccessful. These *Rhodopseudomonas* associates appear to be consistently essential to mycelial life and development. In the same way, repeated subculturing of isolated *Rhodopseudomonas* induced a rapid decline and loss of isolates. There appears to be a reciprocal dependency for long-lasting in vitro growth of both associates.

## Conclusions

In the production of *Tuber*-inoculated plantlets for truffle producers, the use of mycelial cultures is possible but limited by both the availability of fungal cultures and by the mass production of fungal inoculants. This work shows the constant occurrence of *Rhodopseudomonas* associates in the production of *Tuber* mycelium. The availability of both mating types among these mycelial cultures is also of major interest as the occurrence of two compatible mating types is essential to *Tuber* fructification. The marketing of *Tuber*-associated plants, estimated to involve over 500,000 plants per year, could benefit from re-considering the use of mycelial inoculants (instead of applying, at a rate of at least 1 g per plant, crushed truffle fructifications whose market price is about 1000 euros per kilogram) based on the combination of both mating types and their associated *Rhodopseudomonas.* Such practices would allow a better mastering of inoculant quality and consistency, and possibly later on improve and accelerate field fructification of black truffles.

## Additional file

10.1186/s40064-016-2756-6 View of the agarose gel of PCR products obtained with both pairs of primers specific for each mating type on some of the *Tuber* mycelial cultures. Samples MelBal3, TBRS and Mel14 without amplification with both pairs of primers on this gel were successfully amplified elsewhere with *MAT1*-*2*-*1* for the two first and *MAT1*-*1*-*1* for the last one.
