# Supplementary material for: Bacteria of the genus Rhodopseudomonas (Bradyrhizobiaceae): obligate symbionts in mycelial cultures of the black truffles Tuber melanosporum and Tuber brumale
Source: Springerplus. 2016 Jul 15;5(1):1085. doi: 10.1186/s40064-016-2756-6 (PMC4947074; doi:10.1186/s40064-016-2756-6)
Supplement: Supplementary file 1 — 10.1186/s40064-016-2756-6 View of the agarose gel of PCR products obtained with both pairs of primers specific for each mating type on some of the Tuber mycelial cultures. Samples MelBal3, TBRS and Mel14 without amplification with both pairs of primers on this gel were successfully amplified elsewhere with MAT1-2-1 for the two first and MAT1-1-1 for the last one. [file 40064_2016_2756_MOESM1_ESM.pptx]

## Slide 1
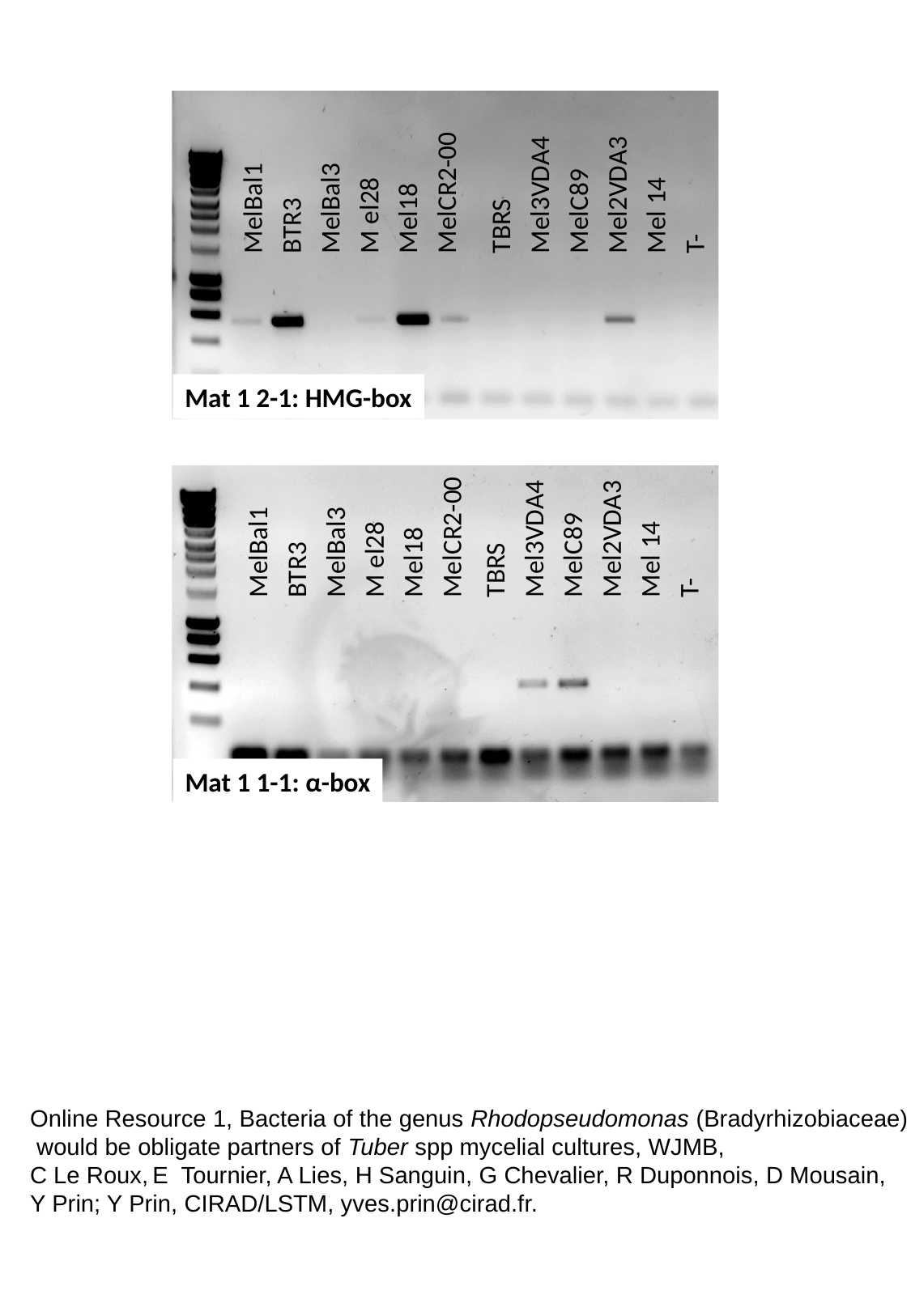

TBRS
Mel3VDA4
MelC89
Mel2VDA3
Mel 14
T-
MelBal1
BTR3
MelBal3
M el28
Mel18
MelCR2-00
Mat 1 2-1: HMG-box
TBRS
Mel3VDA4
MelC89
Mel2VDA3
Mel 14
T-
MelBal1
BTR3
MelBal3
M el28
Mel18
MelCR2-00
Mat 1 1-1: α-box
Online Resource 1, Bacteria of the genus Rhodopseudomonas (Bradyrhizobiaceae)
 would be obligate partners of Tuber spp mycelial cultures, WJMB,
C Le Roux, E Tournier, A Lies, H Sanguin, G Chevalier, R Duponnois, D Mousain,
Y Prin; Y Prin, CIRAD/LSTM, yves.prin@cirad.fr.
